# Supplementary material for: Contribution of the α5 nAChR Subunit and α5SNP to Nicotine-Induced Proliferation and Migration of Human Cancer Cells
Source: Cells. 2023 Aug 4;12(15):2000. doi: 10.3390/cells12152000 (PMC10417634; doi:10.3390/cells12152000)
Supplement: Supplementary file 1 [file cells-12-02000-s001.zip › cells-2502288-supplementary.pdf]

cells

MDPI

Article

Contribution of the α5 nAChR Subunit and α5SNP to Nicotine-Induced Proliferation and Migration of Human Cancer Cells

Irida Papapostolou, Daniela Ross-Kaschitza, Florian Bochen, Christine Peinelt and Maria Constanza Maldifassi *

Institute of Biochemistry and Molecular Medicine, University of Bern, 3012 Bern, Switzerland;
* Correspondence: maria.maldifassi@unibe.ch

Supplementary Figures

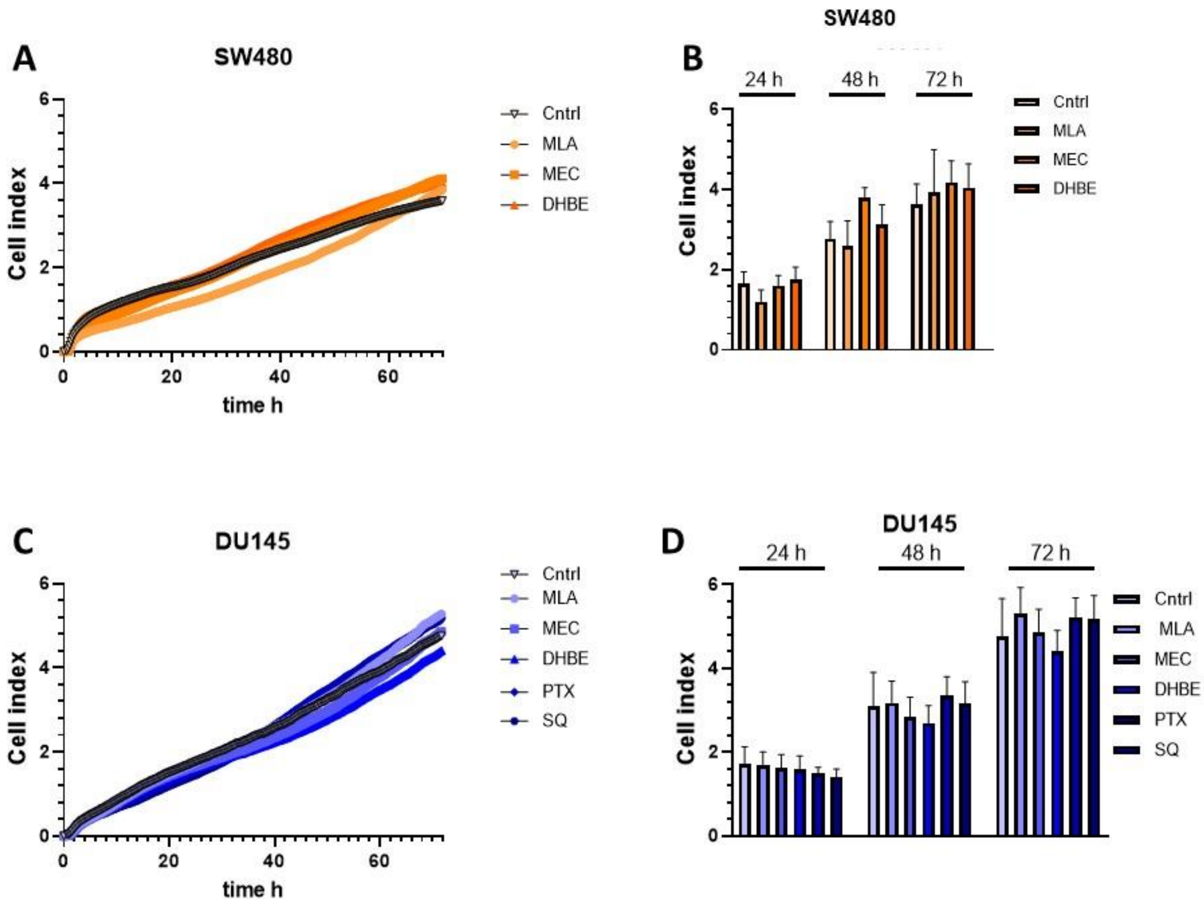

Figure S1. Cellular proliferation index of SW480, and DU145 human cancer cell lines in the presence of diverse inhibitors. (**A**) SW480 cells were treated with the inhibitors MEC, MLA, or DHBE. Proliferation cell index is plotted versus time. (**B**) Bar diagram of data (mean ± SEM) at 24, 48, and 72 h from the experiment in A. (**C**) DU145 cells were treated with the inhibitors MEC, MLA, DHBE, SQ, or PTX. Proliferation cell index is plotted versus time. (**D**) Bar diagram of data (mean ± SEM) at 24, 48, and 72 h from the experiment in B. Measurements were made using 3 independent experiments performed in triplicate. Statistical significance was analyzed using Kruskal–Wallis test for non-parametric data with GraphPad Prism (GraphPad 9.1.1 Software) software. A p-value of <0.05 was considered significant.

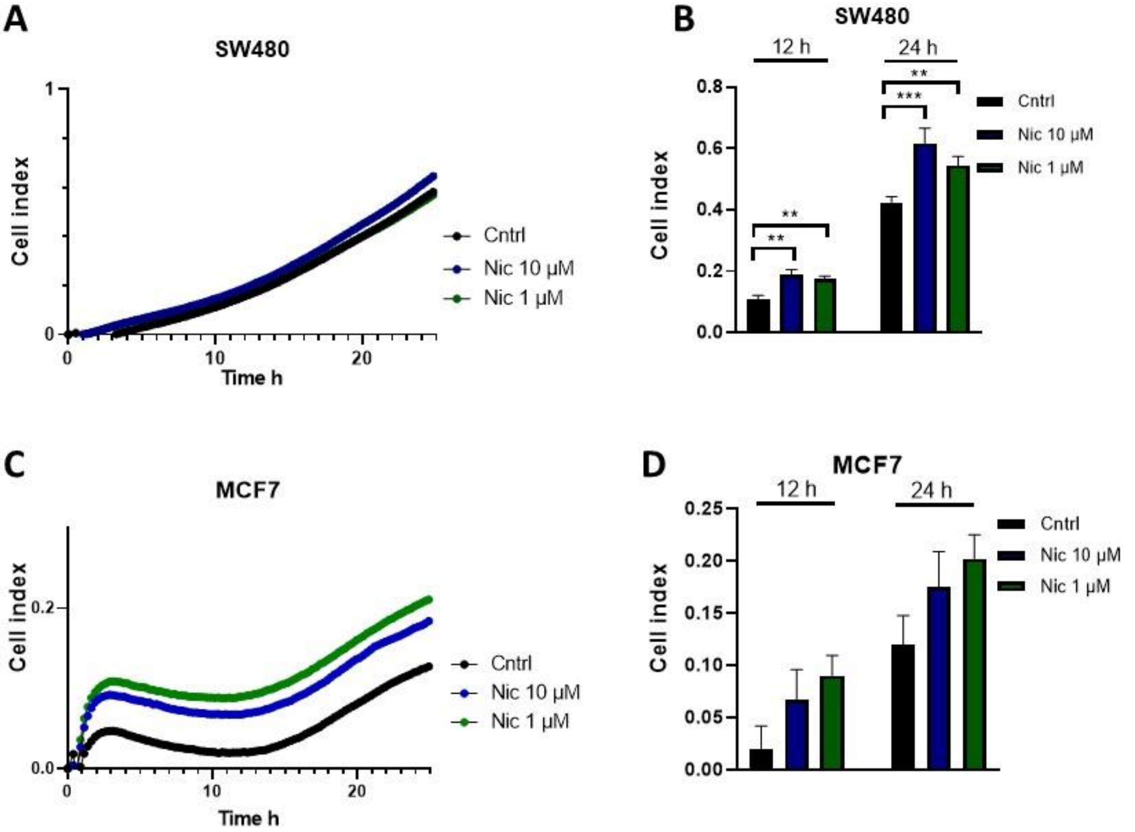

Figure S2. Cell index migration in SW480 and MCF7cells. Analysis was performed using the label-free impedance-based migration assay (xCELLigence). (**A**) SW480 cells were treated with 1 μM, or 10 μM nicotine. Migration cell index is plotted versus time. (**B**) Bar diagram of data (mean ± SEM) at 12 and 24 h from the experiment in A. (**C**) MCF7 cells were treated with 1 μM, or 10 μM nicotine. Migration cell index is plotted versus time. (**D**) Bar diagram of data (mean ± SEM) at 12-24 h from the experiment in C. Measurements were made using 4-5 independent experiments performed in triplicate. Statistical significance was analyzed using Kruskal–Wallis test for non-parametric data with GraphPad Prism (GraphPad 9.1.1 Software) software. A p-value of <0.05 was considered significant. * * $p \leq 0.001$, * ** $p \leq 0.0001$.

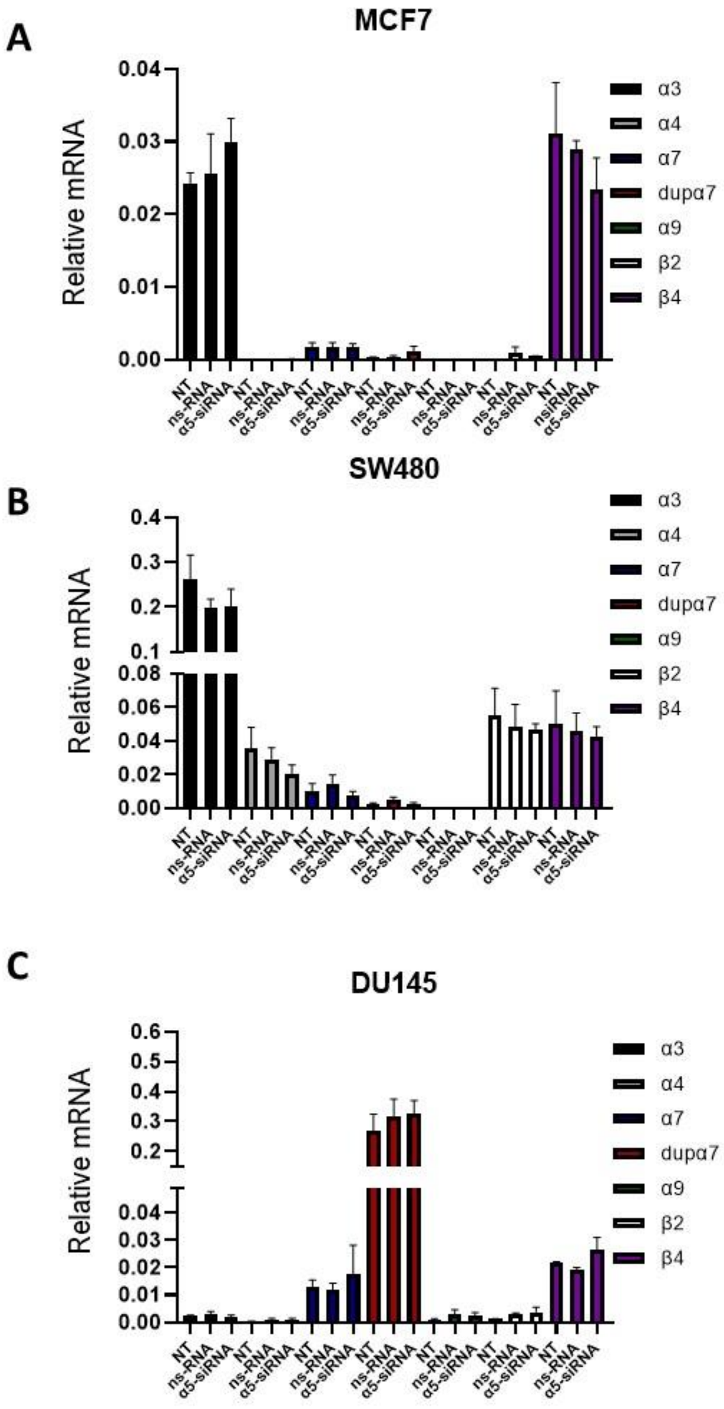

Figure S3. The silencing of α5 in the human cancer cell lines (**A**) MCF7, (**B**) SW480, and (**C**) DU145 does not affect the expression at the mRNA level of other nAChRs subunits. Subunits analyzed: α3, α4, α7, dupα7, α9, β, and β4. The gene expression profile was investigated by means of qPCR analysis. Bar graphs show the relative expression ± SEM of each subunit normalized to endogenous TATA-binding protein expression. Measurements were made using 3-5 independent experiments performed in triplicate.

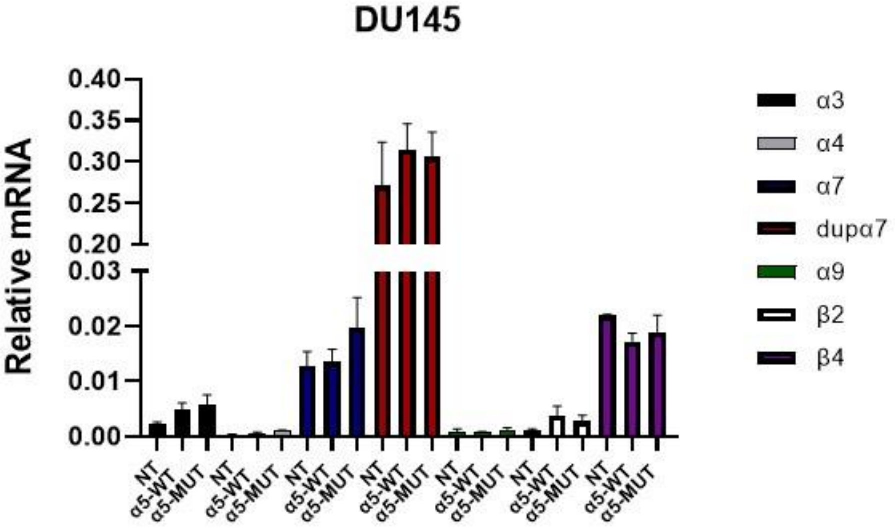

Figure S4. Overexpression of the WT or MUT version of the α5 nAChRs subunit does not affect the expression at the mRNA level of other nAChRs subunits. The human prostate cancer cell line DU145 was transfected with either the wild type α5 construct (α5.pcDNA3.1/V5, α5-WT) or with its mutated version α5D398N (α5D398N.pcDNA3.1/V5, α5-MUT), and the gene expression profile of other subunits was investigated by means of qPCR. Subunits analyzed: α3, α4, α7, dupα7, α9, β, and β4. Bar graphs show the relative expression ± SEM of each subunit normalized to endogenous TATA-binding protein expression. Measurements were made using 3-5 independent experiments performed in triplicate.
